# Characteristics of air quality and sources affecting high levels of PM_10_ and PM_2.5_ in Poland, Upper Silesia urban area

**DOI:** 10.1007/s10661-018-6797-x

**Published:** 2018-08-14

**Authors:** Joanna Kobza, Mariusz Geremek, Lechosław Dul

**Affiliations:** 1 Department of Public Health, School of Public Health in Bytom, Medical University of Silesia in Katowice, Piekarska 18, 41-902 Bytom, Poland; 2 Department of Epidemiology and Biostatistics, School of Public Health in Bytom, Medical University of Silesia in Katowice, Bytom, Poland

**Keywords:** PM_10_, PM_2.5_, Air pollution, Population health, Environmental policy

## Abstract

The reports concerning air quality published by WHO and EEA showed that 33 out of 50 cities with highest concentration of particulate matter (PM)_2.5_ in UE are located in Poland. Various investigations identify main health outcomes to be consistently related to PM_10_ and PM_2.5_. Increased concentration of PM is responsible for 47.3 thousands of premature deaths every year in Poland. The objective of this study was the measurement-based assessment for determining whether the concentrations of PM_10_ and PM_2.5_ are within admissible limits or exceeded in Silesia Province. The data provided by the Voivodship Inspectorate for Environmental Protection in Katowice was used in the analysis. The measurements were made in years 2009–2016 for PM_2.5_ and 2014–2017 for PM_10_ in three measurement stations: two in Katowice (capital of Silesia Province) and one in Żory. The increase in the number of excessive levels of average daily PM_10_ concentration in year 2017 were observed in all three measurement stations, both for the acceptable level, information and alarm level, with lack or singular excessive levels in the previous years. The increase in average annual PM_2.5_ concentrations in year 2016 was also observed, as compared to the previous year in all three measurement stations. The highest pollution is observed in winter. The main cause of exceeded acceptable PM concentrations in Poland is household heating systems, boilers and furnaces burnt with coal or wood, and chimneys. In Silesian Province, the air quality is poor and has deteriorated over the last year.

**Electronic supplementary material:**

The online version of this article (10.1007/s10661-018-6797-x) contains supplementary material, which is available to authorized users.

## Introduction

In the recent years, the highest annual average concentration of particulate matter (PM)_10_ and PM_2.5_ particles in Europe has been observed in the countries located in the East-Central Europe, mainly in Poland. The report published by World Health Organization (WHO) showed that 33 out of 50 cities with highest concentration of PM_2.5_ in European Union (UE) are located in Poland (WHO 2016a). The highest concentrations in excess were found both in large cities, e.g., Cracow, Katowice, and Gliwice, as well as in smaller ones, such as Żywiec, Pszczyna, Rybnik, and Wodzisław (which are in the first five cities), or in middle-sized cities such as Przemyśl and Nowy Sącz. Difficulties with maintaining standards are also experienced by such countries as Bulgaria, Slovakia, and Croatia. A high number of stations in which excessive levels were noted are also in the north of Italy (WHO 2016a; European Environment Agency, 2017).

Air pollution is the main environmental health risk affecting human health (Samoli et al. 2005; Fenger 2009; Anderson 2009; Chang et al. 2015; Li et al. 2016); thus, air quality and its health impact are major public health issues. One of the key indicators concerning air quality monitoring and urban air pollution is the concentration of the suspended PM. PM exposure limitation goals for the protection of human health are included in EU directives (EC 1999, 2008) and numerous WHO documents and guidelines (WHO 1987, 2000, 2006, 2012, 2013).

Particulate matter (PM) is defined as a widespread microscopic air pollutant consisting of solid and liquid particles suspended in the air atmosphere. PM may be dispersed through the air from natural sources (desert dust particles, sea-salt aerosols, wild-land fires) or combustion processes, industrial activities, and communal heating (EEA 2012; OECD 2001). PM includes ions of metals and heavy metals (e.g., potassium, sodium, calcium, magnesium, cadmium, copper, nickel, vanadium, and zinc), sulfates, nitrates, ammonium, and other organic and inorganic chemical compounds as well as allergens and microbial compounds. Primary particles are released directly into the atmosphere; secondary particles are formed by the transformation of the precursors (WHO 2013a).

PM with aerodynamic diameter larger than 10 μm have a relatively high rate of descent in the atmosphere. Therefore, atmospheric aerosols consisting of particulates greater than 10 μm rarely occur far from the source of emissions; thus, their impact on human health is lower. Aerosols dominated by the mass concentration of particles with a diameter smaller than 10 μm (PM_10_), and with a diameter smaller than 2.5 μm (PM_2.5_), have most significant influence on human health, because these particles can penetrate through the respiratory system (WHO 2013b).

## Aim

The objective of this study was the measurement-based assessment for determining whether the concentrations of PM_10_ and PM_2.5_ are within admissible limits or exceeded in Silesia Province. Data was also analyzed to develop key trends from the period 2014–2017 for PM_10_ and from the period 2009–2016 for PM_2.5_.

## Methodology

The data provided by the Voivodship Inspectorate for Environmental Protection in Katowice, collected in the scope of National Environmental Monitoring, was used in the analysis. The received data constitutes the result of 24-h concentrations of PM_2.5_ and PM_10_ particles in suspension in selected air monitoring stations in Katowice (capital of Silesia Province) and Żory. The measurements were made in years 2009–2017 for PM_2.5_ and 2014–2017 for PM_10_. The studies of analyzed suspended particles in selected air monitoring stations are conducted with referential methods specified in appendix no. 6 of the regulation of the Minister of Environment (J. Laws 2012, No. 217, item 1032) on the assessment of levels of substances in the air. The descriptions of measurement methods applied for registration of PM_2.5_ and PM_10_ particles in suspension are presented below, in part on the measurement stations. The values of concentration of PM_2.5_ particles in suspension for the averaging period of 1 year (averaged per year) were calculated as average arithmetic values of 24-h concentrations of the analyzed particles in suspension for a given year. The relative differences in annual concentrations of the analyzed PM_2.5_ particles in suspension in years 2009–2016 for selected measurement stations were calculated according to the following formula:

PM_2.5_: An arithmetic mean of concentration of PM_2.5_ particles in suspension calculated on the basis of average daily values for the averaging period of 1 year;
PD: Acceptable level of concentration of PM_2.5_ particles in suspension for the averaging period of 1 year for year 2015 was 25 μg/m^3^, whereas it has been equal to 20 μg/m^3^ since 2016, with achievement date in year 2020.

For the purpose of more precise and comprehensive analysis of concentrations of PM_2.5_ and PM_10_, the established levels (standards) of concentrations of analyzed particles in suspension were applied. Under the regulation of the Minister of Environment (J. Laws 2012, No. 217, item 1031), the definition of the levels of concentrations of studied particles in suspension used in this paper is the following:

Acceptable level—the level of substance in the air established on the basis of scientific knowledge in order to avoid, prevent, or limit harmful impact on the human health and environment as a whole, which must be achieved on a specified date, and upon such date, it must not be exceeded.

PM_2.5_—for the averaging period of 1 calendar year, the acceptable level was 25 μg/m^3^ with the date of achievement of this value by the concentrations of the analyzed particles in suspension until 2015 and 20 μg/m^3^ for the averaging period of 1 calendar year with the date of achievement of this value by the concentrations of the analyzed particles in suspension until 2020.

Information level—the level of substance in the air above which there is a threat to human health arising from short-term exposure of especially sensitive groups of people to the impact of pollution and, in the case of which, immediate and relevant information is required.

PM_10_—for the 24-h period of averaging measurement results, the information level for the analyzed particles in suspension is equal to 200 μg/m^3^ (the threshold value for informing the society about the risk of exceeding the alarm level for PM_10_).

Alarm level—the level of substance in the air above which there is a threat to health of the entire community arising from short-term exposure to the impact of pollution and, in the case of which, EU member states take immediate actions.

PM_10_—for the 24-h period of averaging measurement results, the alarm level for the analyzed particles in suspension is equal to 300 μg/m^3^.

## Description of measurement points

The measurements were carried out in three measurement stations belonging to Regional Environmental Protection Inspectorate in Katowice.

The first station was located in Katowice, 40–844, Kossutha Street 6. The measurements were carried out from 2014/01/01 to 2017/07/31 for PM_10_ and from 2009/01/01 to 2016/12/31 for PM_2.5_. Measurement type: automatic and manual. Name: SL09KA. International code: PL0008A. Start of measurements: PM_10_—2005-01-01, PM_2.5_—2008-04-01. Cod position: SlKatoKossut-PM_10_—24 h and SlKatoKossut-PM_2.5_—24 h. Method name: gravimetric analysis, LVS—automatic filter change 2.3 m^3^/h. Instrument name: TECORA, model Charlie TCR for PM_10_; ATMOSERVICE PNS3D15/LVS3D for PM_2.5_. Type: urban background station. Measurement zone: Silesian agglomeration. Measurement target: human health protection.

The second station was located in Katowice, Plebiscytowa Street/A4. The measurements were carried out from 2014/01/01 to 2017/07/31 for PM_10_ and from 2011/01/01 to 2016/12/31 for PM_2.5_. Measurement type: automatic, manual, and passive. Name: SL18KA. International code: PL0567A. Start of measurements: PM_10_—2011-01-01, PM_2.5_—2011-01-01. Cod position: SlKatoPlebA4-PM_10_—24 h; SlKatoPlebA4-PM_2.5_—24 h. Method name: gravimetric analysis, LVS—automatic filter change 2.3 m^3^/h. Instrument name: MCZ Umwelttechnik Micro PNS LVS17 for PM_10_; MCZ Umwelttechnik MicroPNS LVS16 for PM_2.5_. Urban communication station. Measurement zone: Silesian agglomeration. Measurement target: human health protection.

The third station was located in Żory, gen. Władysława Sikorskiego 52. The measurements were carried out from 2014/01/01 to 2017/07/31 for PM_10_ and from 2009/01/01 to 2016/12/31 for PM_2.5_. Measurement type: automatic and manual. Name: SL24ZO. International code: PL0489A. Start of measurements: PM_10_—2010-04-07, PM_2.5_—2008-08-19. Cod position: SlZorySikor2-PM_10_—24 h; SlZorySikor2-PM_2.5_—24 h. Method name: gravimetric analysis, LVS—automatic filter change 2.3 m^3^/h. Instrument name: ATMOSERVICE PNS3D15/LVS3D for PM_10_ and PM_2.5_. Type: urban background station. Measurement zone: Rybnik and Jastrzębie agglomeration. Measurement target: human health protection (Map 1).

## Health risk

Increased concentrations of PM_10_ and PM_2.5_ pose a real health risk to the local population. WHO estimated that in 2012, one out of nine deaths was due to the air pollution, and ambient air pollution caused 3 million of those deaths worldwide (WHO 2016b). It is estimated that all-cause daily mortality increases by 0.2–0.6% per 10 μg/m^3^ of PM_10_ and long-term exposure to PM_2.5_ is associated with increase in the long-term risk of cardiopulmonary mortality by 6–13% per 10 μg/m^3^ of PM_2.5_ (WHO 2013a). PM_2.5_ pollution is one of the leading causes of death and disability worldwide resulting in significant health expenditures (WHO 2015). Both short-term exposure (hours, days) on high levels of PM and long-term (months, years) exposure on moderate concentrations of PM have negative influence on human respiratory and cardiovascular systems (Kelly and Fussell 2015).

Multiple studies confirm that PM_2.5_ and PM_10_ can affect lung growth and development in children and adolescents, number of medical visits, and hospital emergency admissions due to the asthma, respiratory symptoms, and upper and lower respiratory tract disorders (Brown et al. 2013; Linares and Diaz 2010; Praznikar and Praznikar 2012; WHO 2013).

High extent of PM_2.5_ could be connected with increased level of AC133+ stem cells in peripheral blood, which may be an early indicator of the cardiovascular system damage (De Jarnett et al. 2015). There are also associations between exposure to air pollutants and hypertension, myocardial infarction, heart failure hospitalizations, and mortality. Anoop et al. estimated that reducing median daily PM_2.5_ concentrations by a mean of 3.9 μg/m^3^ would prevent 7978 heart failure hospitalizations in the USA and would be associated with savings of 307 million USD per year (Anoop et al. 2013). According to Brook et al., short-term exposure to PM_2.5_ (from hours to weeks) can provoke mortality connected with cardiovascular diseases and a long-term (several years of exposure) increases the risk of cardiovascular mortality and reduces life expectancy for several months to a few years. Consequently, the reductions in PM levels are connected with decreases of cardiovascular mortality (Brook et al. 2010). There is also an association between PM_2.5_ exposure and hospital admissions for both ischemic and hemorrhagic stroke (Szu-Ying et al. 2014). The association between PM_2.5_ exposure and hospital admissions for stroke has also been found (Leiva et al. 2013). Increased level of PM_2.5_ particle exposure in the atmosphere should be also considered as a risk factor of premature birth (Malley et al. 2017; Liu et al. 2017).

## WHO and EPA guidelines

According to the guidelines of WHO, the daily concentration of PM_2.5_ should not exceed 25 μg/m^3^ (and not more often than 3 days in a year) and the average annual concentration should not exceed 10 μg/m^3^, whereas daily concentration of PM_2.5_ should not exceed 50 μg/m^3^ and annual concentration should not be higher than 20 μg/m^3^ (WHO 2006). WHO guidelines are stricter than those applicable in EU, like the national law.

Environmental Protection Agency (EPA) in USA is responsible for setting National Ambient Air Quality Standards for key pollutants considered dangerous to general population and the environment; periodically, they are reviewed and may be revised (EPA 2012). The Clean Air Act, amended in 1990, requires identifies two types of national ambient air quality standards: “primary standards provide public health protection, including protecting the health of high-risk populations such as asthmatics, children, and the elderly. Secondary standards provide public welfare protection, including protection against decreased visibility and damage to animals, crops, vegetation, and buildings” (Clean Air Act 1990).

## European and national policy

The European Union has only established the acceptable level for PM_10_ and PM_2.5_, respectively, for PM_10_—50 μg/m^3^ (daily) and 40 μg/m^3^ (average annual) as well as for PM_2.5_—25 μg/m^3^ (average annual) (CAFE 2008). A daily standard for PM_2.5_ has not been established either in the EU regulations or in the Polish law. The EU regulations do not specify the information and alarm level of PM even though it is the case for other substances.

In Poland, the maximum allowable level of average annual concentration of PM_10_ is 40 μg/m^3^ (this level can be exceeded up to 35 times per year), while the maximum allowable level of 24-h mean is 50 μg/m^3^. The maximum allowable level of average annual concentration of PM_2.5_ is 25 μg/m^3^ (to achieve until 1.01.2015) and 20 μg/m^3^ (to achieve until 1.01.2020) (J. Laws 2012, No. 217, item 1031).

As it has been presented above, according to national standards, three levels relating to PM_10_ can be distinguished:

- Acceptable level (daily—50 μg/m^3^), it means that the air quality is not good, but does not result in serious effects on the human health;
- Information level (daily)—200 μg/m^3^, it means that the situation is bad and it is necessary to limit outdoor activity since the standard has been exceeded four times; and
- Alarm level (daily)—300 μg/m^3^, it means that the situation is very bad, the standard has been exceeded six times, and it is absolutely necessary to limit staying outside and it is best to stay home, especially for people with diseases (Table 1).

**Table 1:** PM_10_ and PM_2.5_ concentration standards (in μg/m^3^), based on national and EU legislation, WHO, and EPA recommendation

PM_10_ and PM_2.5_ concentration admissible limits	Time	WHO	EPA	EU	Poland
PM_10_	24-h mean	50 μg/m^3^	150 μg/m^3^, primary and secondary, not to be exceeded more than once per year on average over 3 years	50 μg/m^3^, not to be exceeded more than 35 times a calendar year	50 μg/m^3^, not to be exceeded more than 35 times a calendar year
	Annual mean	20 μg/m^3^	–	40 μg/m^3^	40 μg/m^3^
PM_2.5_	24-h mean	25 μg/m^3^, not to be exceeded more than three times a calendar year	35 μg/m^3^, primary and secondary, averaged over 3 years	–	–
	Annual mean	10 μg/m^3^	12.0 μg/m^3^, primary averaged over 3 years	25 μg/m^3^	25 μg/m^3^
			15.0 μg/m^3^, secondary		Target value 20 μg/m^3^

## Results

### PM_10_ concentrations

The presented radar diagrams include average daily value of PM_10_ concentrations exceeding the acceptable level (50 μg/m^3^) in specified years for selected measurement stations. Only dates for PM_10_ exceeding 150 μg/m^3^ are presented in diagrams for better clarity. With the purpose of more comprehensive (more detailed) analysis of distributions of concentrations of studied particles in suspension, so-called information level (200 μg/m^3^) and alarm level (300 μg/m^3^) are marked in the diagrams.

The analysis of diagrams shows a characteristic distribution of the values of analyzed concentrations of PM_10_ in specific years for the selected measurement stations. In the period of January–February 2017, it was reported the highest number of PM_10_ concentrations above the information level. Also, for the period January–February 2017, the highest values of average daily concentrations of analyzed particles in suspension were noted as compared to the previous years.

**Fig. 1:**
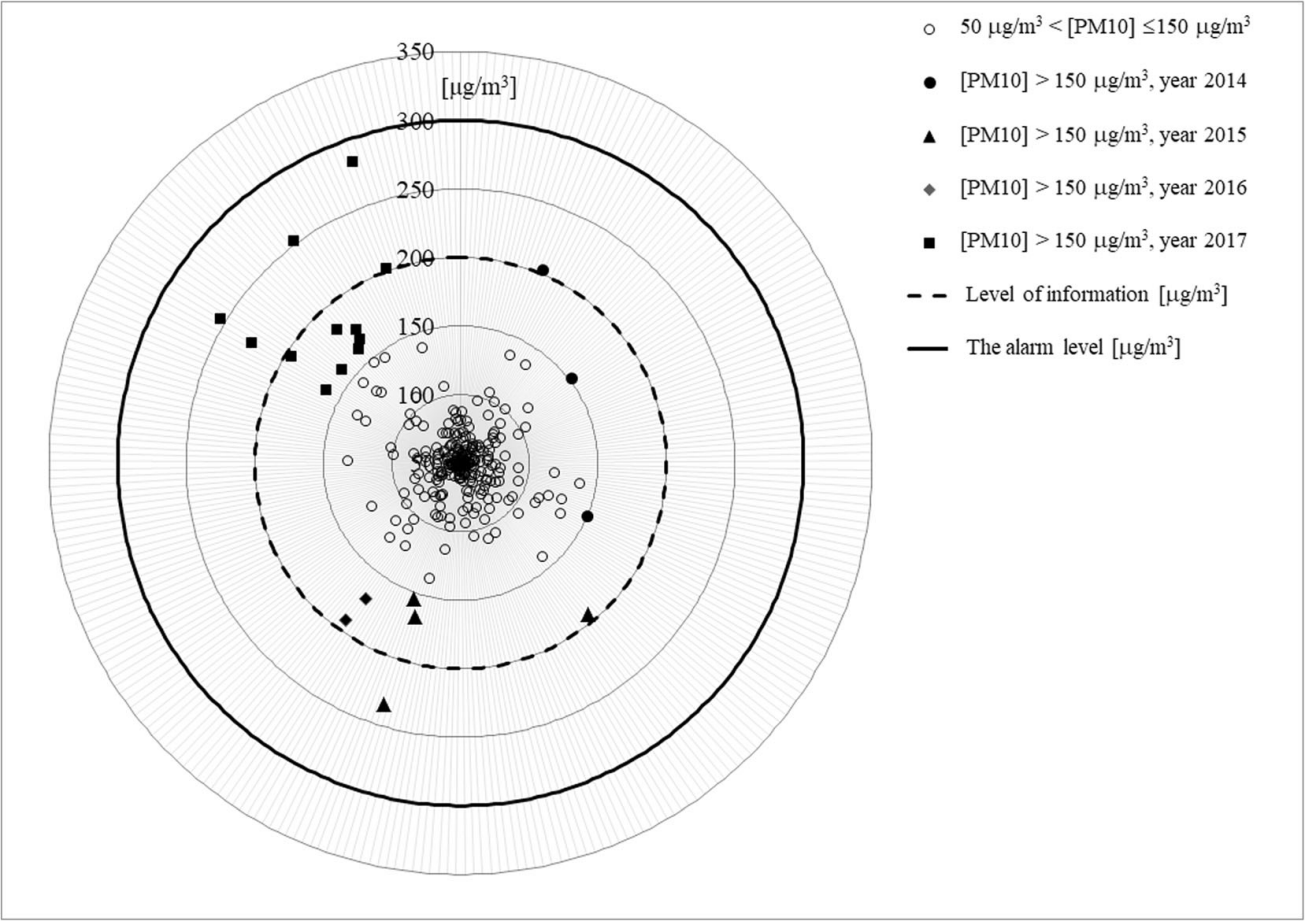
Measurement station in Katowice—PL0008A

**Fig. 2:**
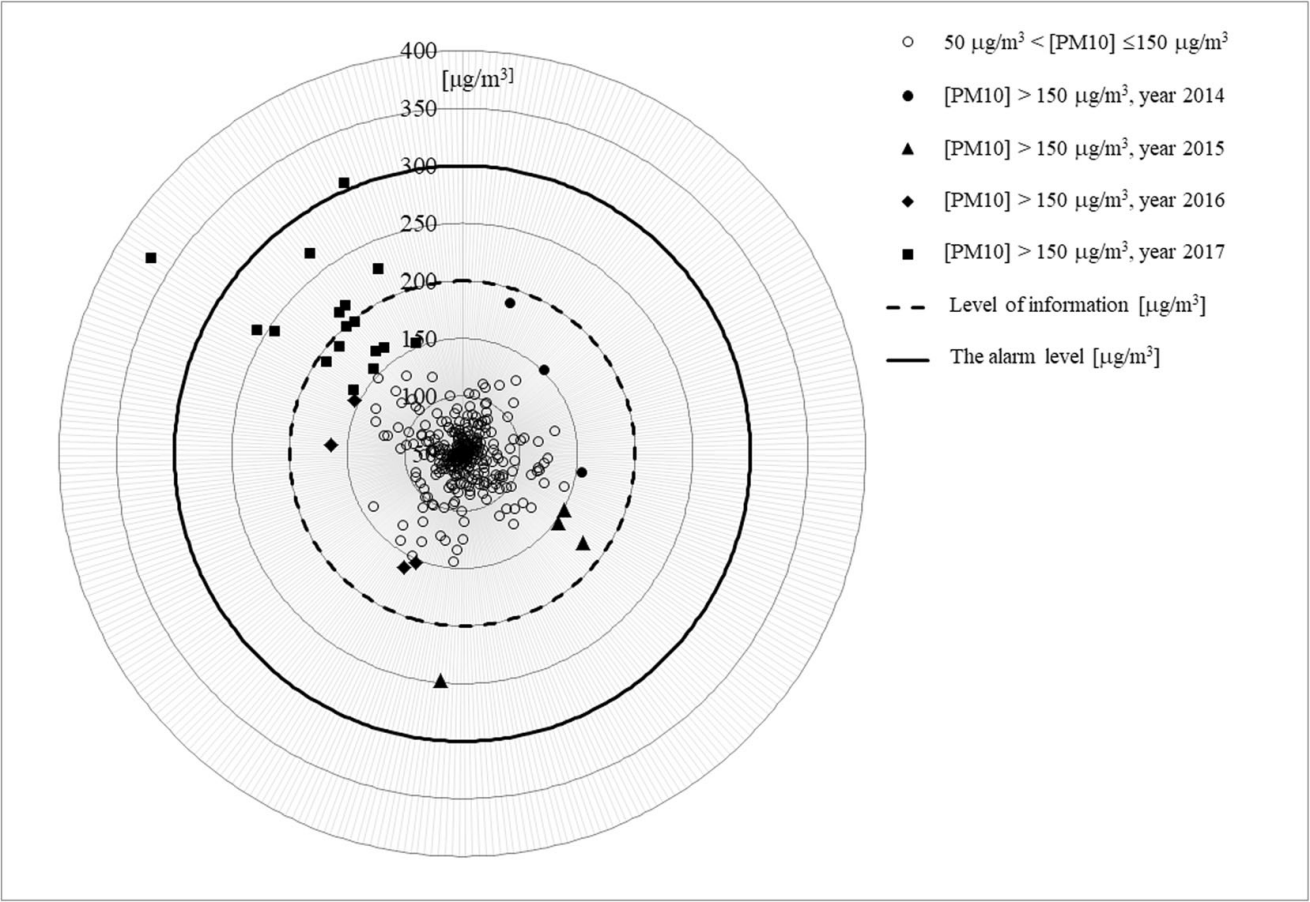
Measurement station in Katowice—PL0567A

**Fig. 3:**
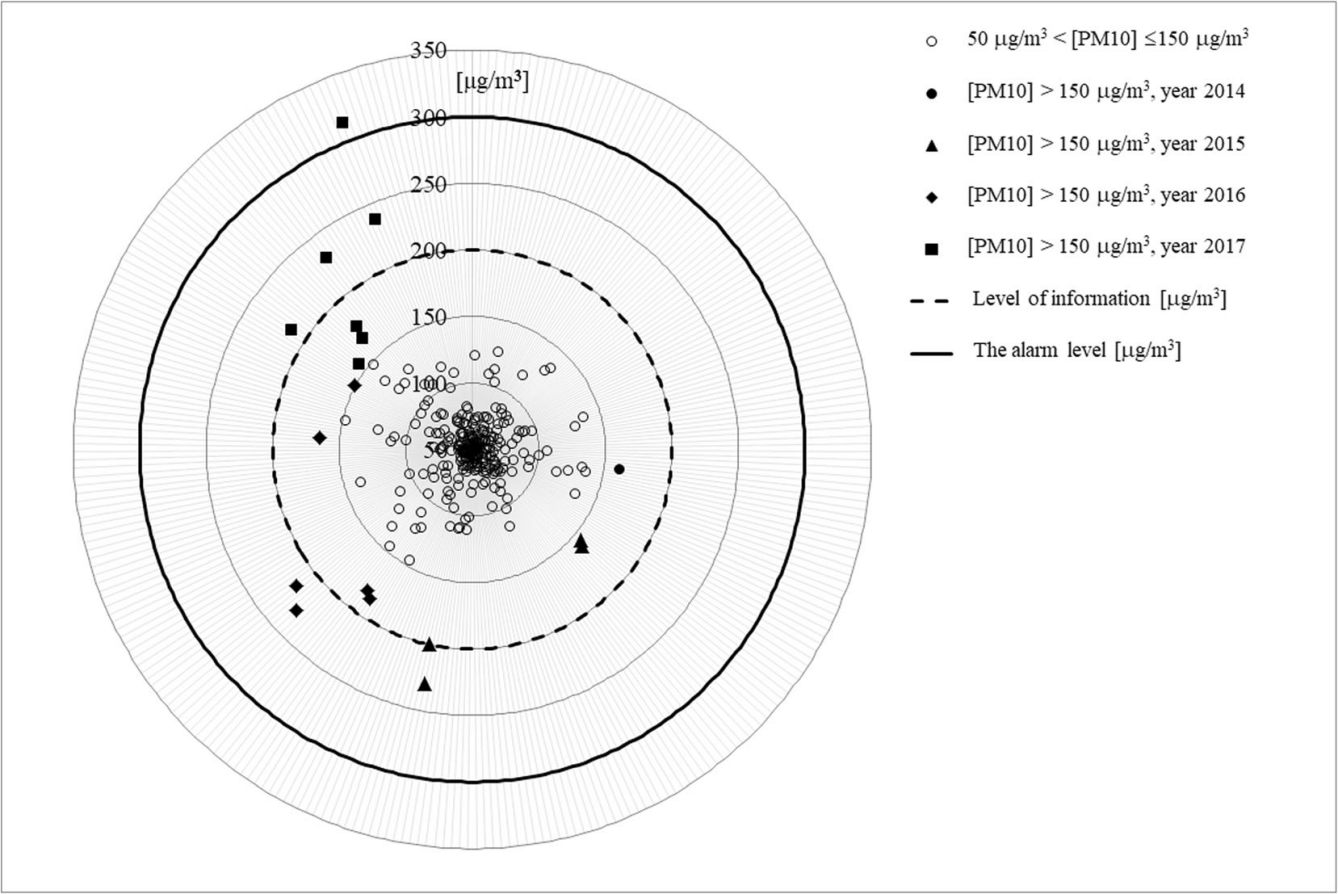
Measurement station in Żory—PL0489A

The obtained results mean that the condition of air pollution with PM_10_ particles in suspension at the present time has not decreased, but it is even possible to state that it is subject to exacerbation (Figs. 1, 2, and 3 and Table 2).

**Table 2:** PM_10_ concentrations, the information level exceedances, above 200 μg/m^3^, in period 2014–2017, data obtained from three automatic stations of State Environmental Monitoring in the Silesian Voivodship (Upper Silesian urban area)

Measurement stations	Year	PM_10_—the information level exceedances above 200 μg/m^3^	Date
PL0008A	2014	203.0 μg/m^3^	04 February
Katowice	2015	234.7 μg/m^3^	05 November
	2016	Not reported	–
	2017	226.1 μg/m^3^	08 January
	2017	254.8 μg/m^3^	10 January
	2017	253.2 μg/m^3^	01 February
	2017	202.2 μg/m^3^	14 February
	2017	284.1 μg/m^3^	15 February
PL0567A	2014	Not reported	–
Katowice	2015	248.1 μg/m^3^	05 November
	2016	Brak	–
	2017	258.0 μg/m^3^	08 January
	2017	369.5 μg/m^3^	09 January
	2017	244.6 μg/m^3^	10 Jan.
	2017	212.8 μg/m^3^	28 Jan.
	2017	214.3 μg/m^3^	31 January
	2017	268.2 μg/m^3^	01 February
	2017	226.7 μg/m^3^	14 February
	2017	306.2 μg/m^3^	25 February
PL0489A	2014	Not reported	–
Żory	2015	229.5 μg/m^3^	05 November
	2016	229.3 μg/m^3^	19 January
	2016	217.2 μg/m^3^	23 January
	2017	212.7 μg/m^3^	01 January
	2017	230.4 μg/m^3^	01 February
	2017	237.1 μg/m^3^	14 February
	2017	304.2 μg/m^3^	15 February

For comparison of the previous years, we also presented the data from period 2006–2013. It is worth noting that the maximum levels in winter months were much lower than over the past 4 years (Table 3).

**Table 3:** PM_10_ concentrations (maximum and minimum level during each year) in period 2006–2013, data obtained from automatic station of State Environmental Monitoring in the Silesian Voivodship—PL0008A (Upper Silesian urban area)

Year	PM_10_ concentration maximum level in μg/m^3^	PM_10_ concentration minimum level in μg/m^3^
2006	118 January	37 June
2007	64 March	25 January
2008	54 December	30 June
2009	68 December	23 June
2010	122 December	30 January
2011	99 November	23 July
2012	121 February	25 June, July
2013	65 January	25 June

### PM_2.5_ concentrations

The radar diagrams for PM_2.5_ show the values of average annual concentrations of analyzed particles in suspension in specific years for selected measurement stations. The acceptable level of PM_2.5_ concentration is presented in the diagrams for the studied particles in suspension, which was 25 μg/m^3^ until 2015 and has been equal to 20 μg/m^3^ since 2016 with achievement date in 2020.

**Fig. 4:**
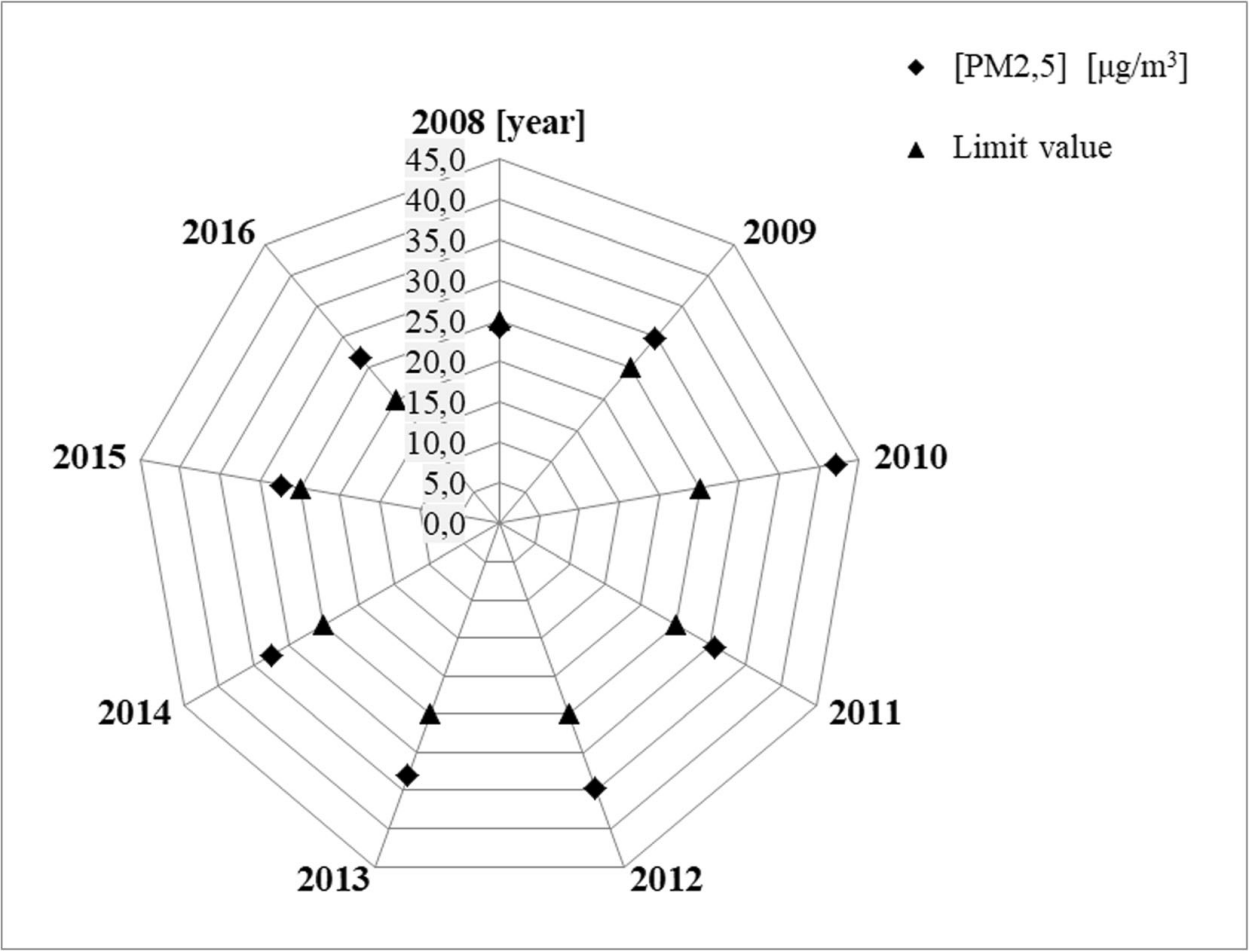
Measurement station in Katowice—PL0008A

**Fig. 5:**
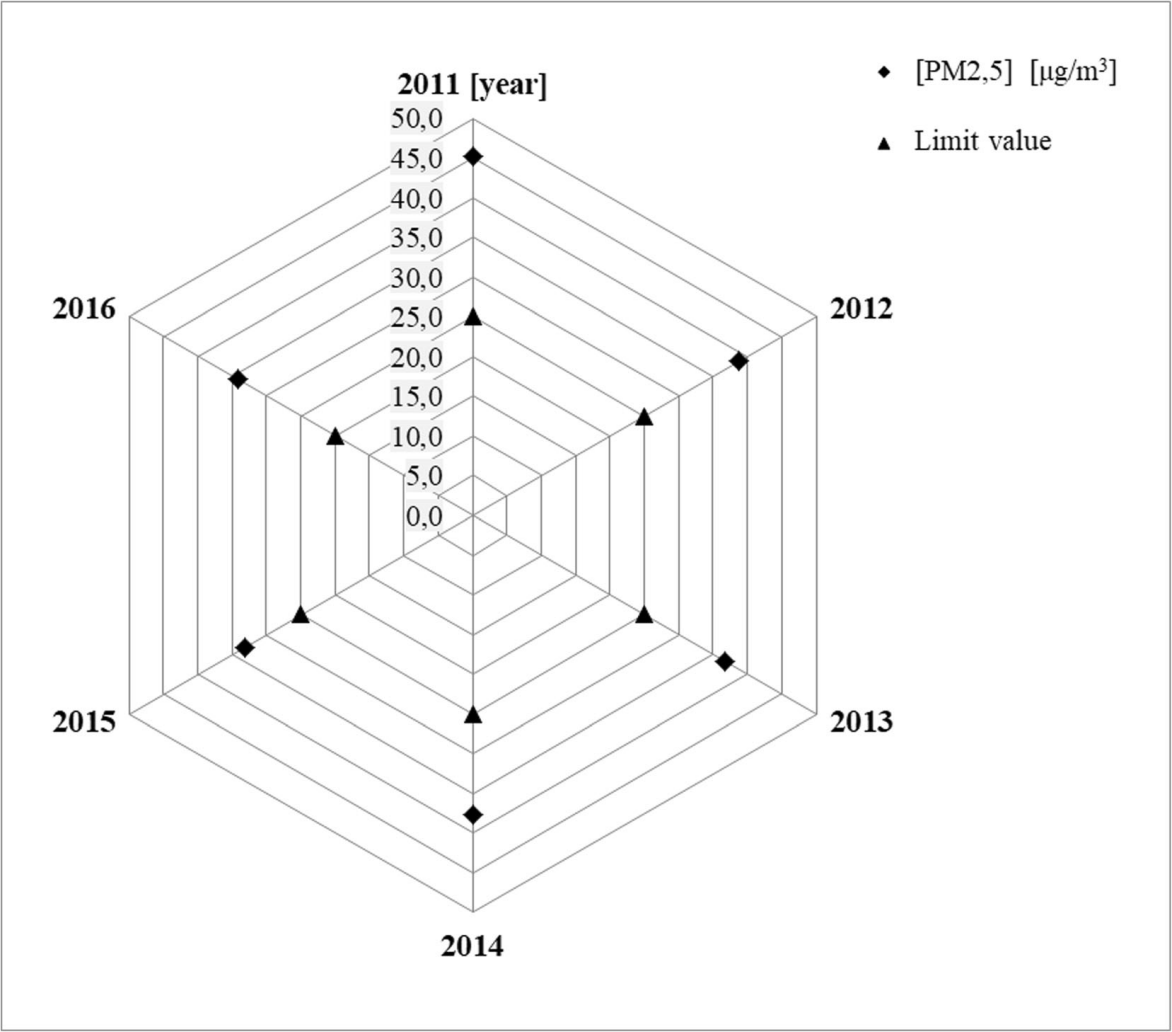
Measurement station in Katowice—PL0567A

**Fig. 6:**
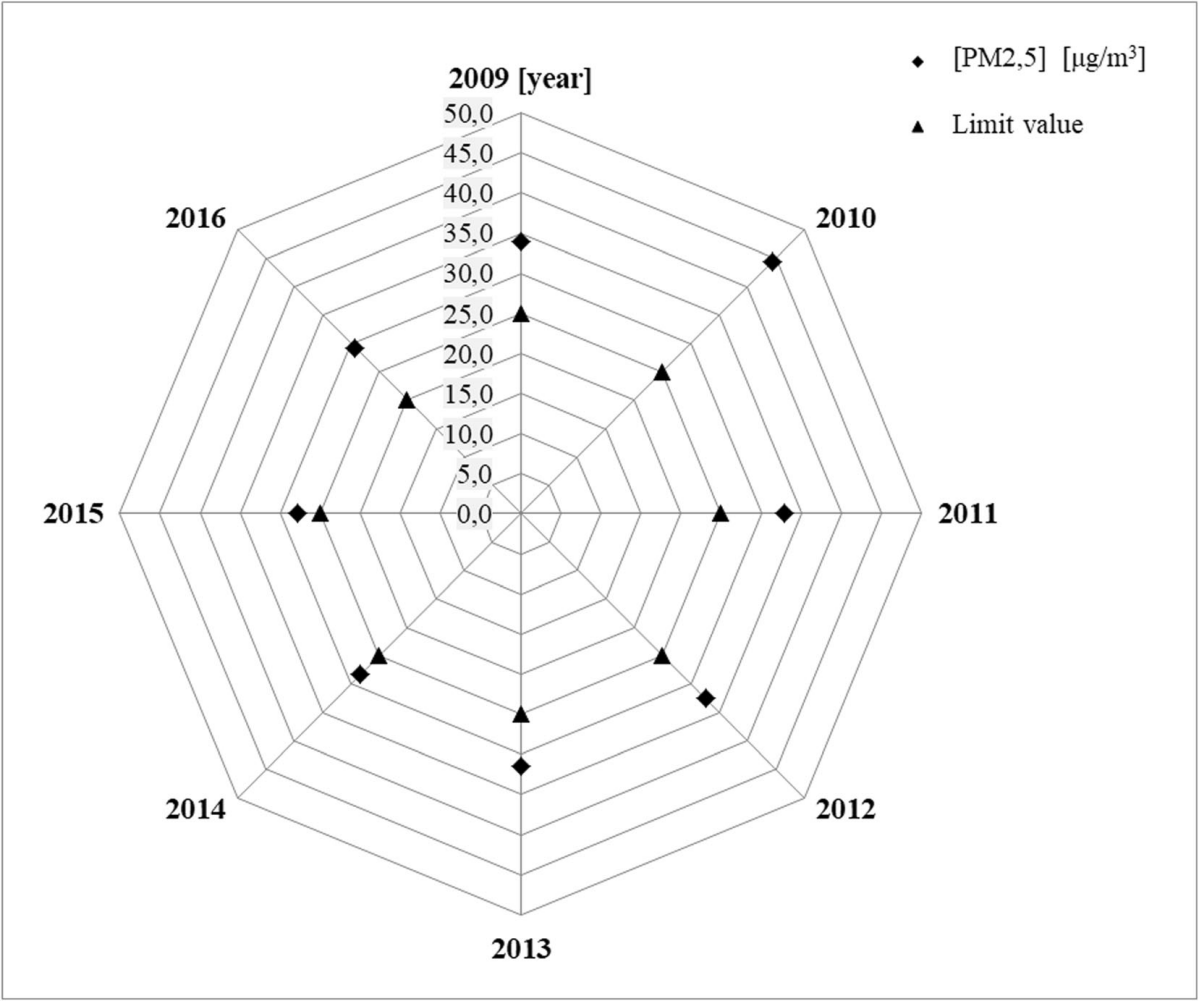
Measurement station in Żory—PL0489A

The analysis of shapes of distributions of average annual concentrations of PM_2.5_ conducted on the basis of prepared radar diagrams allowed for formulation of a very important information. The average annual concentrations of PM_2.5_ particles in suspension exceed the acceptable values in selected measurement stations in years 2009–2016. The calculated relative differences in annual concentrations of the analyzed PM_2.5_ particles in suspension (%) in years 2009–2016 for selected measurement stations clearly indicate that air pollution with PM_2.5_ particles in suspension in year 2016 did not decrease, but was even observed to increase. The clear increase of PM_2.5_ concentration in year 2016, as compared to the previous years, only in the measurement station in Katowice, Kossutha Street 6 (PL0008A), results from the change in the acceptable level. For other selected measurement stations, the clear increase of PM_2.5_ in year 2016, as compared to the previous years, does not depend on the change of the acceptable level. The change of the acceptable level only influences the size of observed PM_2.5_ (%) (Figs. 4, 5, and 6 and Table 4).

**Table 4:** The relative differences in annual concentrations of the analyzed PM_2.5_ particles in suspension in years 2009–2016 for selected measurement stations, for acceptable level 25 μg/m^3^, and in 2016 additionally for level 20 μg/m^3^

Measurement stations	Years	PM_2.5_ (%) PD-acceptable level—25 μg/m^3^ in 2016 also for new level—20 μg/m
PL0008A	2009	19.4
Katowice	2010	68.5
	2011	22.4
	2012	38.5
	2013	32.1
	2014	30.0
	2015	9.1
	2016	33.1 (6.4) PD—20 μg/m^3^
PL0567A	2011	81.0
Katowice	2012	55.0
	2013	47.0
	2014	50.9
	2015	32.5
	2016	71.1 (36.9) PD—20 μg/m^3^
PL0489A	2009	35.6
Żory	2010	77.4
	2011	31.9
	2012	30.3
	2013	25.8
	2014	13.5
	2015	11.1
	2016	46.4 (17.1) PD—20 μg/m^3^

Calculated according to the following formula: PM_2.5_ − an arithmetic mean of concentration of PM_2.5_ particles in suspension calculated on the basis of average daily values for the averaging period of 1 year and PD − acceptable level of concentration of PM_2.5_ particles in suspension for the averaging period of 1 year for year 2015 was 25 μg/m_3_, whereas it has been equal to 20 μg/m_3_ since 2016, with achievement date in year 2020)

It is essential that the highest concentrations of PM_10_ and PM_2.5_, both in terms of annual mean and number of days on which daily concentration standard was exceeded, are noted not only in large cities but also in contrast to the majority of EU member states, small towns without industry, or busy road traffic. In addition, the highest pollution concentrations are observed in winter. It results from the fact that the main cause of exceeded acceptable PM concentrations in Poland is burning coal and/or biomass in residential boiler plants as well as heating buildings individually, household heating systems, boilers and furnaces burnt with coal or wood, and chimneys (The National Centre for Emissions Management 2017) (Table 5).

**Table 5:** Sources of emissions and main causes of PM_10_ and PM_2.5_ permissible concentration exceedances in Poland, based on The National Centre for Emissions Management 2016 and Chief Inspectorate of Environmental Protection 2015 (Rok 2017)

Air pollutant	Main sources of emission	Main causes of permissible concentration exceedances in Poland
PM_10_	Communal heating—38% Road transport—9% Commercial power industry—9%	Communal heating—85% Intensive traffic of vehicles—9%
PM_2.5_	Communal heating—40% Road transport—13% Commercial power industry—10%	Communal heating—89% Intensive traffic of vehicles—8%

The worst situation is in the area of southern Poland as it is a site where the following factors accumulate: high density of detached residential development, common use of coal in household boilers, industrial emission, and land shape contributing to accumulation of pollution (Chief Inspectorate of Environmental Protection 2017).

## Discussion

Air in Poland is one of the most polluted in the entire European Union, and the standards included in the EU and Polish law concerning the air quality have not been followed over the last years. Air pollution (mainly PM_10_ and PM_2.5_) is responsible for 47.3 thousands of premature deaths every year in Poland (Supreme Audit Office 2014). It has been noted that the situation is subject to exacerbation in this scope (Sobolewski 2016). As far as the air pollution emitted by industry and energetics has been significantly limited due to implementation of the requirements for this sector at the level of EU regulations (Kobza et al. 2016), there is a lack of effective regulations concerning the heating systems used in households, i.e., furnaces and boilers burnt with solid fuels or chimneys (Supreme Audit Office 2016). Although Poland has adopted several vehicle emission control policies over the past decades (Kobza and Geremek 2017), there are still no key legal solutions in the scope of transport which would allow to limit the use of cars which pollute the air most.

The establishment of the National Program of Air Protection (ME 2015) and adoption of the so-called anti-smog law seem to be not enough. The amendment of the Environmental protection law (a so-called anti-smog law) finally adopted in October 2015 allowed the voivodship self-governments in the entire Poland to introduce prohibitions of burning coal in the communes and heating only with high-quality coal or modern furnaces. Two voivodships have introduced it and the next three are preparing it. Supreme Audit Office highlighted in their reports that Ministry of Environment in Poland should introduce emission limits for a new household coal-fired boiler systems and implement quality standards for solid fuels (Supreme Audit Office 2014; Supreme Audit Office 2016).

Despite the obligation not to exceed annual and daily acceptable values for PM_10_ particles in suspension came into force on 1 January 2005 and the situation of exceeding daily acceptable PM_10_ values in Poland did not improve upon 2013, in connection with the aforementioned, it was decided to initiate the procedure on the infringements of CAFE Directive. In December 2015, the European Commission filed a complaint to the Court of Justice of the European Union for the second time, in which it included the following irregularities:

- Exceeding the daily acceptable values for PM_10_ particles in 35 air quality zones in the territory of the state since 2007,
- Lack of adoption of appropriate actions in air protection programs aiming to achieve the shortest period of occurrence of exceeded acceptable PM_10_ values, and
- Inappropriate transposition of CAFE directive to the Polish legal system.

With low effectiveness of current actions in the scope of air quality improvement, the estimates of external costs of air pollution, especially health (WHO 2016b) and economic costs (OECD 2015; World Bank and Institute for Health Metrics and Evaluation 2016), can provide important arguments in discussion. The results of scientific studies constitute an essential argument for experts, politicians, and society with the purpose of taking actions to improve the situation. More determined and consistent policy of air pollution reduction in Poland would have had non-negligible public health profits.

## Conclusion

The results of this study provide the evidence that in the territory of the Silesian voivodship, the air quality is poor and has deteriorated over the course of the last year. The increases in the number of excessive levels of average daily PM_10_ concentration in year 2017 were observed in all three measurement stations, both for the acceptable level, information and alarm level, with lack or singular excessive levels in the previous years. In addition, the increase in average annual PM_2.5_ concentrations in year 2016 was also observed as compared to the previous year.

## Electronic supplementary material

ESM 1 Map of air pollution monitoring sites in Katowice and Żory in the Upper Silesian agglomeration (JPG 183 kb)
