# Supplementary figures and images for: Characteristics of air quality and sources affecting high levels of PM10 and PM2.5 in Poland, Upper Silesia urban area
Source: Environ Monit Assess. 2018 Aug 14;190(9):515. doi: 10.1007/s10661-018-6797-x (PMC6096885; doi:10.1007/s10661-018-6797-x)

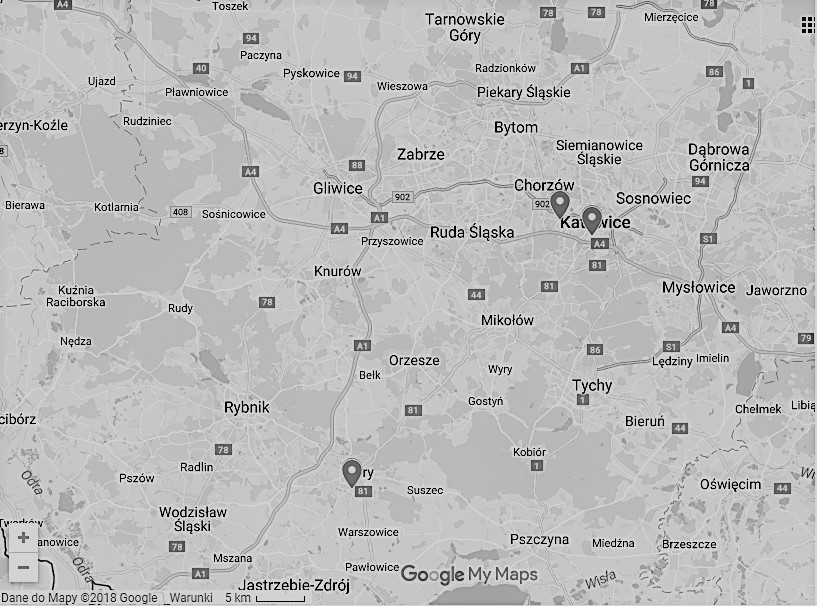

Supplement: Supplementary file 1 — Map of air pollution monitoring sites in Katowice and Żory in the Upper Silesian agglomeration (JPG 183 kb) [file 10661_2018_6797_MOESM1_ESM.jpg]
